# A Novel Corn-Expressed Phytase Improves Daily Weight Gain, Protein Efficiency Ratio and Nutrients Digestibility and Alters Fecal Microbiota in Pigs Fed with Very Low Protein Diets

**DOI:** 10.3390/ani10101926

**Published:** 2020-10-20

**Authors:** Cedrick N. Shili, Jonathan N. Broomhead, Shelby C. Spring, Mike B. Lanahan, Adel Pezeshki

**Affiliations:** 1 Department of Animal and Food Sciences, Oklahoma State University, Stillwater, OK 74078, USA; cedrick.shili@okstate.edu (C.N.S.); scspring2@wisc.edu (S.C.S.); 2 Agrivida, Woburn, MA 01801, USA; poultrynut49@gmail.com (J.N.B.); mike.lanahan@agrivida.com (M.B.L.)

**Keywords:** low-protein diets, low calcium/phosphorous diets, corn-expressed phytase, growth, nutrients digestibility, bone characteristics, fecal microbiota, pigs

## Abstract

**Simple Summary:**

Feeding pigs with very low crude protein (CP) diets with reduced calcium (Ca) and phosphorous (P) may be an effective strategy to decrease the nutrient excretion; however, this practice can negatively impact the growth performance of animals. Thus, there is an urgent need to improve the utilization of nitrogen, Ca and P in pigs. This study aimed to assess the effect of a novel corn-expressed phytase on growth performance, nutrients digestibility, gut microbial population, bone mineral density and content and blood metabolites in nursery pigs fed with low-CP, -Ca, and -P diets. Supplementation of very low protein diets with a corn-expressed phytase decreased the negative effects of these diets on average daily gain and protein efficiency ratio of pigs, increased the digestibility of Ca and P regardless of the levels of these minerals in the diet, improved bone characteristics and altered the fecal bacterial composition. This study suggests that corn-expressed phytase can be potentially useful for improving the growth performance of weaned pigs fed with low protein diets and should be considered for sustainable swine production.

**Abstract:**

The objective of this study was to assess the effect of a novel corn-expressed phytase (CEP) on growth, nutrients digestibility, bone characteristics and fecal microbiota of pigs fed with very low-protein, -calcium (Ca) and -phosphorous (P) diets. Forty-eight barrows were subjected to 6 groups for 4 weeks: positive control-adequate protein (PC), negative control-reduced protein (NC), NC + low-dose CEP, i.e., 2000 FTU/kg (LD), NC + high-dose CEP, i.e., 4000 FTU/kg (HD), LD with 0.12% unit reduced Ca and 0.15% unit reduced available P (LDR), and HD with 0.12% unit reduced Ca and 0.15% unit reduced available P (HDR). Compared to NC, LD and HDR had a higher average daily gain (ADG) and gain:protein ratio (G:P), HD and HDR had greater apparent fecal digestibility of Ca and P and bone mineral density and LDR and HDR had lower serum osteocalcin. The feces of LD was enriched in Lachnospiraceae, while the HD had a higher abundance of *Succinvibrio* and LDR had a higher abundance of *Bifidobacterium* and Actinobacteria. In conclusion, supplementation of protein-restricted diets with a CEP decreased their negative effects on ADG and G:P ratio, increased the digestibility of Ca and P regardless of the levels of these minerals in the diet, improved bone characteristics and produced differential effects on fecal bacterial population.

## 1. Introduction

The sustainability of the swine industry is challenged by increased feed cost and environmental concerns associated with excessive excretion of pollutants such as nitrogen (N) and phosphorus (P) from swine production [1,2]. Feeding pigs with low protein diets with reduced P may help decrease the nutrients excretion [3,4]. Slightly low protein diets, supplemented with limiting amino acids (i.e., lysine, methionine, threonine, and tryptophan), can be used to decrease the excretion of N and feed cost and mitigate the incidence of diarrhea after weaning without negative impact on performance or feed efficiency in pigs [5,6,7,8]. Severe reduction of dietary CP, i.e., > 25%, may decrease the nutrients excretion more than slight protein restriction; however, very low protein diets while supplemented with limiting amino acids, decrease the feed efficiency and growth performance of pigs [9,10,11]. Similarly, diets with low P:calcium (Ca) ratio compromise the growth measures such as daily gain and bone development and increase the feed conversion rate in pigs [12]. Thus, there is a need to develop strategies for improving the N and P utilization in pigs fed with very low-protein and -P diets to minimize their negative effects on animals’ growth.

Exogenous phytase has been widely used in the swine diet to improve the utilization of nutrients through the breakdown of insoluble complexes formed between nutrients and phytate (*myo*-inositol hexaphosphate) [2,13]. The phytate is a polyanionic molecule and unavailable form of phosphorus that can chelate divalent cations such as Ca with a high capacity and create mineral-phytate complexes [2]. Similar insoluble complexes are formed between phytate and proteins [14], inhibiting the activity of proteolytic enzymes [15], and reducing the utilization of protein. The most common type of exogenous phytase used in the swine diet is microbial phytase. There is a plethora of evidence that digestibility and utilization of P and Ca are increased in pigs when microbial phytase was supplemented in their diets formulated with adequate Ca and P [16,17,18,19] or reduced Ca- and P [20,21,22,23,24,25]. However, there are conflicting reports on the effects of microbial phytase on the digestibility of N and amino acids in pigs [26,27]. Some studies have shown promising outcomes on growth performance when microbial phytase was used for pigs receiving amino acids-deficient diets [28,29], but some others have reported no positive effects on the growth of animals [30,31]. 

Microbial phytase is produced by fermentation from yeasts, fungi, and multiple strains of bacteria [32]. In particular, two strains of *Aspergillus* sp., *A. ficuum,* and *A. niger* have been commonly used for commercial production of microbial phytase [32]. An alternative cost-effective strategy for commercial production of phytase is through its expression in transgenic plants that due to their large biomass they can express the transferred genes and produce recombinant phytase extensively [33]. There are limited animal studies to test the efficacy of recombinant phytase as an alternative for microbial phytase. Recently, a novel corn-expressed phytase (CEP) was shown to improve the growth performance, bone characteristics and digestibility of Ca and P in weaned pigs fed with Ca- and P-deficient diets [34,35]. However, it remains to be determined whether the CEP improves the growth performance of young pigs when they are fed with low protein diets. Further, little is known on the mechanisms by which the CEP improves the growth performance of pigs. The beneficial effect of the CEP on the growth performance of pigs has been mainly attributed to improved digestibility of Ca and P [34,35]. There is some evidence that changes in intestinal availability of Ca and P as a result of using microbial phytase can affect the activity of the intestinal microbiota in pigs [36,37]; however, little is known whether dietary supplementation of CEP influences the performance of pigs through changes in fecal microbiota. Further, the effect of CEP on bone mineral density and content and blood metabolites associated with Ca and P metabolism is not known. Therefore, the objective of the current study was to investigate the effect of two levels of a CEP on growth performance, nutrient digestibility, fecal microbiota composition, bone mineral density, and content and blood metabolites associated with Ca and P metabolism in young pigs fed with very low protein diets with reduced Ca and P. 

## 2. Materials and Methods 

### 2.1. Animals, Housing, and Diets

The experimental procedures used during this entire study were performed in accordance with FASS Guide for the Care and Use of Agricultural Animals in Research and Teaching, and all the experimental procedures were approved by the Institutional Animal Care and Use Committee at Oklahoma State University (Animal Care and Use Protocol # AG-17-22). A total of 48 weanlings (three weeks old; 6.3 ± 1.2 kg body weight (BW)) crossbred barrows (Duroc sire line and Large White X Landrace dam) were used (Seaboard, Hennessey, OK, USA). The general animal husbandry procedures were undertaken according to our previous publications [38,39]. Upon arrival, the pigs were group-housed and acclimated to the environment in a controlled temperature and ventilation facility. The temperature was set at 31 °C during the first week and then it was reduced by 1 °C every week. Feed was provided in one-hole stainless steel feeders, and water was provided by cup waterers (Aqua Chief™) with single 1/2″ nipples (Lixit^®^ Nipple Waterer-L-70). Both feed and water were provided *ad libitum*. All pigs used in this study were barrows (male). Following two weeks of adaptation, all pigs were weighed, individually housed in a 60 × 167 cm pen and assigned to one of six groups (n = 8/group) while keeping the mean body weight consistent for all groups (10.2 ± 1.5 kg). Each group then was randomly allotted to one of the dietary treatments including, (1) positive control with normal protein content (PC); (2) negative control with low protein content (NC); (3) NC+ low dose of CEP (LD); (4) NC+ high dose of CEP (HD); (5) LD with reduced Ca and P (LDR); (6) HD with reduced Ca and P (HDR). The ingredients and composition of diets used are given in Table 1. The dose of CEP used for LD and HD diets was 2000 one phytase unit (FTU)/kg and 4000 FTU/kg. The used doses for CEP was chosen based on recent research published on corn-expressed phytase (GraINzyme^®^, Agrivida, Woburn, MA, USA), the same product used in this study, where 4000 FTU/Kg phytase was shown to increase the growth performance of pigs fed with diets with normal protein content [35]. Both LDR and HDR diets had reduced Ca and available P by 0.12 and 0.15 units [35], respectively compared to the rest of the diets (Table 1). All diets were corn-soybean based and prepared according to Nutrient Requirements of Swine-National Research Council [40]. Phase feeding was applied according to requirements of pigs during the nursery period [40] with providing the commercial nursery phase 1 pelleted diet (United Animal Health, Sheridan, IN, USA) for one week (days 1–7), nursery phase 2 diet for two weeks (days 8–21) and nursery phase 3 diet for three weeks (days 22–42). The dietary treatments were fed from day 14 of the study (Table 1). 

All diets were isocaloric with low protein diets being isonitrogenous (Table 1). The desired energy and protein content of the diets were achieved by manipulating the amount of soybean, cornstarch, corn, and limiting amino acids (i.e., lysine, methionine, threonine, and tryptophan). The Ca and P levels in LDR and HDR diets were obtained by reducing the amount of dicalcium phosphate and limestone. All diets contained 0.5% chromium oxide (AquaPhoenix Scientific Inc, Hanover, PA, USA) as an indigestible marker. The CEP used in this study (GraINzyme^®^) was provided by Agrivida Inc. (Woburn, MA, USA, St Louis, MO, USA). The specific activity of GraINzyme^®^ was 3200 FTU/g. 

### 2.2. Growth Performance Parameters 

The individual feed intake and BW were recorded daily and weekly, respectively. The average daily gain (ADG) was calculated by dividing the weight gain of each pig during the experimental period by 28 days (weeks 2–6). The average daily feed intake (ADFI) was calculated by dividing the cumulative feed intake (CFI) of each pig during the treatment feeding period by 28 days (weeks 2–6). Body weight gain (BWG) to feed intake ratio considered as gain to feed ratio (G:F) and BWG to protein intake ratio defined as gain to protein ratio (G:P) were computed by dividing the overall weight gain by cumulative feed intake or cumulative protein intake, respectively. The BWG, CFI, cumulative protein intake (CPI), and G:F and G:P ratios were calculated weekly.

### 2.3. Feed, Fecal, and Blood Samples Collection

Approximately 500 g of feed samples were collected after mixing each diet and stored at −20 °C for further analysis. Fecal samples were collected at weeks 5 and 6 of the study by transferring the pigs to metabolic crates. Each pig was housed in a metabolic crate for two consecutive days with free access to feed and water. Collected fecal samples in plastic bags were pooled and stored at −20 °C for nutrients digestibility tests.

At the end of the study (week 6), blood samples were drawn from the anterior vena cava (jugular) of each pig in the supine position using a 20-gauge vacutainer needle in 10 mL sterile serum tubes (BD, Franklin Lakes, NJ, USA). Blood samples were placed on ice after collection, transferred to the laboratory and centrifuged for 10 min at 4 °C and at 2000 ×*g* to collect the serum. The collected serum was stored at −80 °C until further analysis. 

At week 6 of the study, fresh fecal samples were collected from the rectum of all pigs in the fecal collection tubes (Global Scientific, Wilmington, NC, USA) as previously described [38,39], placed on ice, transferred to the laboratory, and stored at −80 °C for bacterial DNA extraction and sequencing.

### 2.4. Bone Mineral Density and Bone Mineral Content Analysis 

At the end of the study, all pigs were euthanized via CO2 asphyxiation method and the whole carcass of each pig was scanned in ventral position with extended limbs by dual-energy X-ray absorptiometry (DXA) (Hologic, Discovery QDR Series, Bedford, MA, USA) in the same day by the same operator as previously explained [23]. The DXA scanner provided the measurements of bone mineral density (BMD) and bone mineral content (BMC). The standard procedure of the manufacturer was used for calibration of the scanner and to obtain the DXA scans for analysis. Previous studies have shown a high correlation between bone measurements using DXA and bone ash content in pigs suggesting that DXA can be used accurately for evaluation of bone and skeletal status through measuring the mineral content and density in live animals [41].

### 2.5. Proximate Analysis of Feed Samples 

All feed samples were analyzed by ServiTech laboratories (Dodge City, KS, USA). As we previously described [38,39], experimental diets were analyzed for moisture, crude protein, crude fiber, crude fat, nitrogen, Ca, and P, and chromium using official methods of analysis of AOAC [42]. 

### 2.6. Nutrients Apparent Fecal Digestibility 

Fecal samples were analyzed for Ca, P, N, and chromium as indicated above for feed samples by ServiTech (Dodge City, KS, USA). The apparent fecal digestibility (AFD) for Ca, P, and N were calculated for individual animals using the marker method, which is based on the differential concentrations of analyzed chromium (used as an external marker) and the nutrient in feed and feces, according to the following formula: AFD = 100 − (100 × (marker concentration in feed/marker concentration in feces) × (nutrient concentration in feces/nutrient concentration in feed) [43].

### 2.7. Fecal DNA Isolation, Amplicon Sequencing, Sequence Data Analysis, and Taxonomic Classification 

The DNA from fecal samples was isolated using the QIAamp DNA stool mini kit (Qiagen, Inc., Germantown, MD, USA) as we previously described [38,39], and following the instructions of the manufacturer. Isolated DNA samples concentration and quality were determined (Epoch, Biotek; Winooski, VT, USA) and stored at −80 °C. The samples with DNA concentration greater than 6 ng/μL with the OD 260/280 of 1.8–2 were used for PCR amplification and microbial amplicon sequencing (Novogene Corp., Sacramento, CA, USA).

As we previously described [38,39], for amplicon sequencing, the primers 515F (5′-GTGCCAGCMGCCGCGGTAA-3′) and 806R (5′-GGACTACHVGGGTWTCTAAT-3′) and Phusion^®^ High-Fidelity PCR Master Mix (New England Biolabs, Ipswich, MA) were used for amplifying the 16S rRNA V4 region by PCR. The identical volume of PCR products was mixed with 1× loading buffer containing SYBR green and loaded on 2% electrophoresis agarose gel for quality control and quantification. The PCR products were extracted from the agarose gel using GeneJET Gel Extraction Kit (Thermo Scientific, Waltham, MA, USA), and the sequencing library was prepared using NEB Next^®^ Ultra™ DNA Library Prep Kit for Illumina (NEB, San Diego, CA, USA) per manufacturer’s instructions. Index codes were added and the library quality was determined by using the Qubit@2.0 Fluorometer (Thermo Scientific, Waltham, MA, USA) and Agilent Bioanalyzer 2100 system. The library was then sequenced using the Illumina HiSeq 2500 platform (Illumina, Inc.), and 250 bp paired-end raw reads were generated. 

For sequence data analysis, paired-end reads were assigned to samples based on their unique barcode and then the barcode and primer sequence was truncated. Paired-end reads were then merged using FLASH (V1.2.7, http://ccb.jhu.edu/software/FLASH/) [44] to obtain splicing sequences called raw tags. Using specific filtering conditions of QIIME (V1.7.0, http://qiime.org/index.html) [45], quality filtering was performed on the raw tags, which produced high-quality clean tags. To detect chimeric sequences and remove them [46], the tags were compared with the reference database (Gold database, http://drive5.com/uchime/uchime_download.html) using UCHIME algorithm (UCHIME Algorithm, http://www.drive5.com/usearch/manual/uchime_algo.html) [47], and Effective Tags were obtained. The obtained Effective Tags were clustered by Uparse software (Uparse v7.0.100http://drive5.com/uparse/) [48] and assigned to Operational Taxonomic Units (OTU) based on at least 97% similarity. 

For taxonomic classification, OTU representative sequences were classified using the QIIME-based wrapper of the Ribosomal Database Project (Version 2.2, http://sourceforge.net/projects/rdp-classifier/) [49] and naive Bayesian classifier retrained on the Greengenes 16S rRNA gene database (http://greengenes.lbl.gov/cgi-bin/nph-index.cgi) [50], using a 0.80 confidence threshold. Rarefaction curves were generated through a random selection of certain sequencing data. Further, the beta diversity of fecal bacterial communities was assessed by Principal Coordinate Analysis (PCoA) and weighted and unweighted UniFrac methods [51] using QIIME (Version 1.7.0, http://qiime.org/index.html).

### 2.8. Serum Osteocalcin, Calcium, Phosphorus, and Alkaline Phosphatase

Serum osteocalcin concentration was determined using a porcine enzyme-linked immunosorbent assay kit (Cloud-Clone Corp., Katy, TX, USA) according to the manufacturer’s instructions. The absorbance values were measured using a microplate reader (Epoch from Biotek, Winooski, VT, USA) at 450 nm. Serum Ca, P, and alkaline phosphatase (ALP) were analyzed using a chemical chemistry analyzer (CLC 480/BioLis24i, Carolina Liquid Chemistry, Brea, CA, USA). Prior to analyzing the samples, the analyzer was calibrated as instructed by the manufacturer. 

### 2.9. Statistical Analysis 

The sample size was calculated with Al-Therapy Statistics (https://www.ai-therapy.com/psychology-statistics/sample-size-calculator) using data from our previous study [38]. For 8 nursery pigs/group, a difference in BW of 7.90 kg (SD: control diet = 4.27 kg, low protein diet = 4.26 kg) can be detected with 93% power (α = 0.05; effect size = 1.852). Overall growth performance, bone measurements, minerals digestibility, and concentration of serum metabolites and hormones data were analyzed using the univariate ANOVA procedure of SPSS^®^ (IMB SPSS Statistics version 23, Armonk, NY, USA). Repeated measures on weekly growth performance were analyzed with a linear mixed model. Diet, time, and the interaction of diet and time were included in the model as fixed, and the animal was a random variable. Based on the smallest values of fit statistics for corrected Akaike’s Information Criterion and Bayesian Information Criterion, the covariance structure of the repeated measurements for each variable was modeled as either first-order antedependence, autoregressive, heterogeneous autoregressive, compound symmetry, heterogenous compound symmetry, or toeplitz. Means of dietary groups were separated by Tukey’s post hoc analysis. *p* ≤ 0.05 and 0.05 < *p* ≤ 0.1 were considered as statistical significance and trends, respectively. For quantitative analysis of gut microbiota composition within dietary groups, linear discriminant analysis (LDA) with effect size measurements (LEfSe) was used using a tool hosted in the Galaxy (server) instance of Huttenhower lab (https://huttenhower.sph.harvard.edu/galaxy/) and the scores were normalized by log10. The populations with LDA score (log10) > 2 were considered as bacterial with markedly increased numbers. To determine the significantly different beta diversity among dietary groups pairwise, Wilcoxon test was performed with differences being considered significant at *p* value ≤ 0.05.

## 3. Results

### 3.1. Body Weight, Feed Intake, and Feed Efficiency 

The initial BW was not different (*p* = 0.56) among groups, with an average BW of 10.18 kg for experimental groups (Table 2). The overall effect of diet on final BW, ADG, ADFI, G:F, and G:P was significant (*p* ≤ 0.05). Pigs fed positive PC had higher final BW, ADG, ADFI, and G:F than other groups (Table 2). Relative to NC, pigs fed LD and HDR tended to have 26% higher BW. Pigs fed LD and HDR had 84% and 80% higher ADG than the NC, respectively. Further, pigs fed with LDR tended to have a 66% higher ADG than those fed with NC (Table 2). No differences were detected on final BW and ADG when CEP supplemented groups with reduced Ca and P (i.e., LDR and HDR) were compared to the CEP supplemented diets with adequate Ca and P (i.e., LD and HD). Similarly, there were no differences in final BW and ADG when the groups with two doses of CEP (i.e., LD vs. HD or LDR vs. HDR) were compared (Table 2). Except week 1, there was no difference in BWG of PC and LD, positive control had higher BWG compared to all other treatments during the study (Table 3). In the second week, pigs in the HDR group gained 129% more weight than those in the NC group (Table 3).

The ADFI of pigs fed with NC was not different compared to groups fed LD, HD, LDR and HDR (Table 2). The ADFI of LD and HD did not differ compared to LDR and HDR, respectively. Further, the ADFI was not different for LD vs. HD and LDR vs. HDR (Table 2). CFI and CPI were significantly higher for PC pigs than those in other treatments during whole study (Table 3). Pigs fed LD tended to have 25% and 23% higher CFI than NC and LDR, respectively, during the first week of the study (Table 2). 

The LD, LDR, and HDR treatments tended to have a 53%, 61%, and 68% higher G:F than NC (Table 2). The LD also tended to have a 55% higher G:P and HDR had a 74% higher G:P compared to NC. LDR and HDR were not different from LD and HD on their G:F and G:P, respectively (Table 2). Also, when two different doses of CEP used were compared (i.e., LD vs. HD and LDR vs. HDR), no differences in G:F and G:P were detected. In the first week of the study, although NC pigs tended to have lower G:F than those received PC, no differences in G:F was detected when groups LD, HD, LDR, and HDR were compared with PC (Table 3). In week 2, except HDR that was not different from PC, all other groups had lower G:F than PC. In week 3, pigs fed with LD, HD and HDR were not different in G:F from those fed PC. At week 4, although NC had lower G:F than PC, none of the other groups showed a significant difference in G:F compared to PC. Further, LDR and HDR pigs had 156% and 170% higher G:F than those fed with NC. In weeks 1 and 3, no differences in G:P were detected among dietary groups. In week 2, the HDR and in week 4, the LDR and HDR had a 132% and 172% higher G:P than NC, respectively (Table 3).

### 3.2. Bone Mineral Content and Density 

Pigs fed with NC had significantly lower BMD compared to PC; however, none of the CEP supplemented groups were different from PC in BMD (Table 2). Pigs in the LD group tended to have higher BMD and the ones in HD and HDR had significantly higher (17% and 18%) BMD than those in the NC group, respectively. No differences in BMD were detected for: LD vs. HD, LDR vs. HDR, LD vs. LDR and HD vs. HDR. NC and LDR had lower BMC than PC; however, no differences in BMC were detected when LD, HD and HDR were compared to PC. Further, LD had 29% and 27% higher BMC than NC and LDR, respectively. No differences in BMC were detected when LD vs. HD, LDR vs. HDR and HD vs. HDR were compared (Table 2).

### 3.3. Apparent Fecal Digestibility of Calcium, Phosphorus, and Nitrogen 

**Figure 1:**
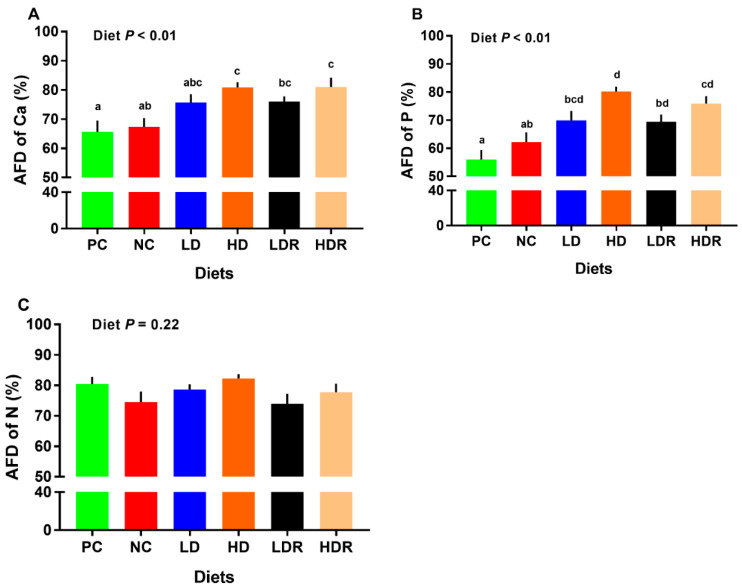
Nutrients’ digestibility in pigs fed with low-protein diets supplemented with a corn-expressed phytase. (**A**) apparent fecal digestibility (AFD) of calcium (Ca), (**B**) AFD of phosphorus (P), (**C**) AFD of nitrogen (N). Pigs are grouped based on their dietary treatments: PC (positive control): normal protein, adequate Ca and available phosphorous (aP), no corn-expressed phytase (CEP) added; NC (negative control): low protein, adequate Ca and aP, no CEP added; LD: NC + CEP added at low dose, i.e., 2000 FTU/kg of diet; HD: NC + CEP added at high dose, i.e., 4000 FTU/kg of diet; LDR: LD with reduced calcium (Ca) and phosphorus (P); HDR: HD with reduced Ca and P. The values are means ± standard errors of means. Different letters in the bar plots indicate significant differences (*p* ≤ 0.05, Tukey’s test). *n =* 8 for each dietary group.

The AFD of Ca was not different between the PC and NC groups. HD, LDR and HDR had higher AFD of Ca than PC (Figure 1A and Table S1). The AFD of Ca for HD and HDR was higher than NC (Figure 1A). The AFD of Ca for Ca- and P-deficient diets supplemented with CEP (i.e., LDR and HDR) was not different compared to that for Ca and P-adequate diets supplemented with CEP (i.e., LD and HD). Further, the AFD of Ca was not different for LD vs. HD and LDR vs. HDR comparisons.

All experimental groups (i.e., LD, HD, LDR, and HDR) had higher AFD of P compared to PC (Figure 1B and Table S1). Also, a significant increase in AFD of P was observed for HD and HDR compared to NC, while the LDR and HDR were not different compared to LD and HD, respectively. Additionally, the AFD of P for LD vs. HD and LDR vs. HDR was not different (Figure 1B). No differences across treatments were detected for AFD of N (Figure 1C and Table S1).

### 3.4. Serum Osteocalcin, Calcium, Phosphorus, and Alkaline Phosphatase 

**Figure 2:**
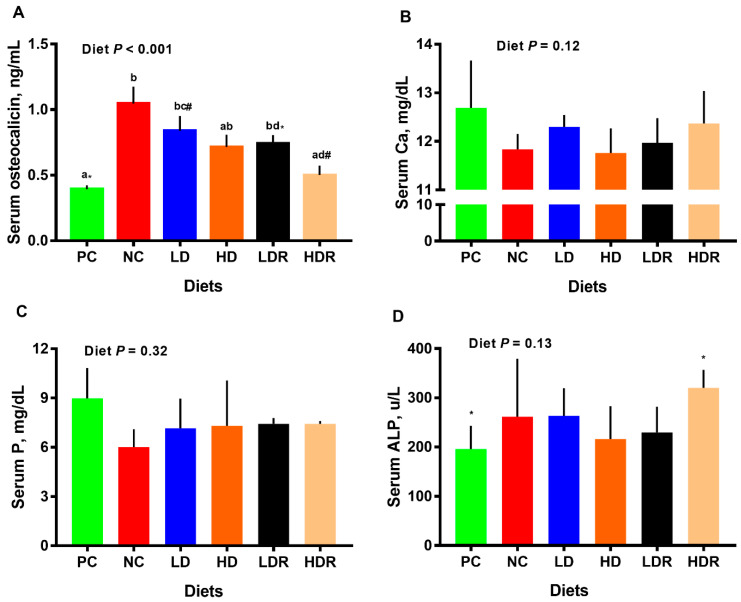
Serum metabolites and hormones in pigs fed with low-protein diets supplemented with a corn-expressed phytase. (**A**) serum osteocalcin, (**B**) serum calcium (Ca), (**C**) serum phosphorus (P), (**D**) serum alkaline phosphatase (ALP). Pigs are grouped based on their dietary treatments: PC (positive control): normal protein, adequate Ca and available phosphorous (aP), no corn-expressed phytase (CEP) added; NC (negative control): low protein, adequate Ca and aP, no CEP added; LD: NC + CEP added at low dose, i.e., 2000 FTU/kg of diet; HD: NC + CEP added at high dose, i.e., 4000 FTU/kg of diet; LDR: LD with reduced calcium (Ca) and phosphorus (P); HDR: HD with reduced Ca and P. The values are means ± standard errors of means. Different letters in the bar plots indicate significant differences (*p* ≤ 0.05, Tukey’s test) and common superscript symbols indicate a trend (0.05 < *p* ≤ 0.1). *n* = 7–8 for each dietary group.

Compared to PC, pigs fed diets with NC and LD had significantly higher (*p* < 0.01) concentration of serum osteocalcin (Figure 2A). Relative to NC, no differences in serum osteocalcin were observed in LD, HD, and LDR, but pigs fed with HDR had significantly lower serum osteocalcin (*p* < 0.01). There was no difference in serum osteocalcin when LD vs. LDR, HD vs. HDR, and LDR vs. HDR were compared (Figure 2A). No differences among dietary groups were detected for serum Ca, P, and ALP concentrations (Figure 2B–D). Serum ALP tended to be higher for HDR relative to PC (Figure 2D).

### 3.5. Fecal Microbiota

As shown in the rarefaction curve analysis, at 30,000 reads and 600–1200 OTU, all fecal samples analyzed reached a stable plateau (Figure S1A,B) suggesting a sufficient sequencing depth for capturing the species richness of the samples assessed.

**Figure 3:**
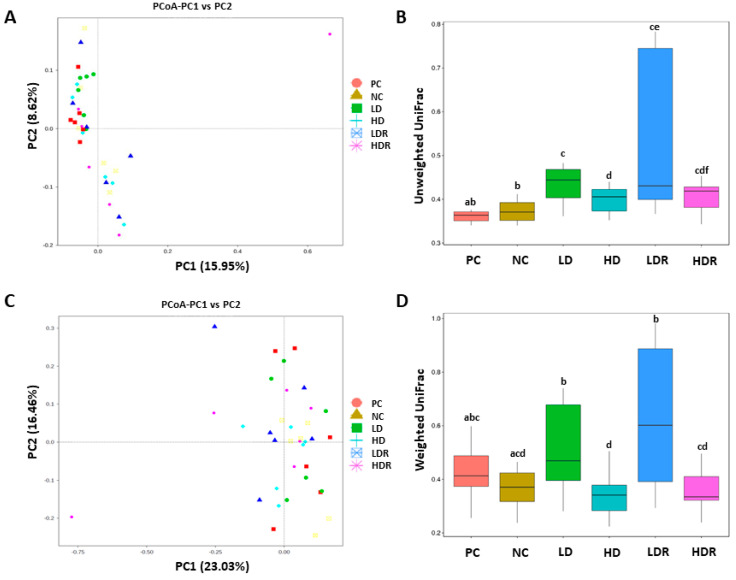
Beta diversity of the fecal bacterial community in pigs fed with low-protein diets supplemented with a corn-expressed phytase. (**A**) Principal Coordinates Analysis (PCoA) of unweighted UniFrac distances showing the diversity of fecal microbiota across individual animals assigned to 6 dietary groups. Each node represents an individual pig, (**B**) Unweighted UniFrac distances shown for each dietary group, (**C**) PCoA of weighted UniFrac distances showing the diversity of fecal microbiota across animals assigned to 6 dietary groups. Each node represents an individual pig, (**D**) Weighted UniFrac distances shown for each dietary group. Pigs are grouped based on their dietary treatments: PC (positive control): normal protein, adequate Ca and available phosphorous (aP), no corn-expressed phytase (CEP) added; NC (negative control): low protein, adequate Ca and aP, no CEP added; LD: NC + CEP added at low dose, i.e., 2000 FTU/kg of diet; HD: NC + CEP added at high dose, i.e., 4000 FTU/kg of diet; LDR: LD with reduced calcium (Ca) and phosphorus (P); HDR: HD with reduced Ca and P. The values are means ± standard errors. Different letters in the box plots indicate significant differences (*p* ≤ 0.05, Wilcoxon test). *n* = 6 for each dietary group.

PCoA and unweighted and weighted UniFrac distances are shown in Figure 3A–D. There was a significant difference in the beta diversity of bacterial communities between CEP supplemented groups (i.e., groups LD, HD, LDR, and HDR) with NC and PC when unweighted UniFrac distances analysis was applied (Figure 3B). No differences in beta diversity were seen for PC vs. NC, LD vs. LDR, HD vs. HDR, and LDR vs. HDR, but there was a difference between LD and HD (Figure 3B). When weighted UniFrac distances analysis was applied, beta diversity was significantly different between HD and PC (Figure 3D). LD and LDR had significantly different beta diversity than NC, but no differences were detected for NC vs. HD and NC vs. HDR. Further, LD and LDR were different when compared to HD and HDR, respectively (Figure 3D).

**Figure 4:**
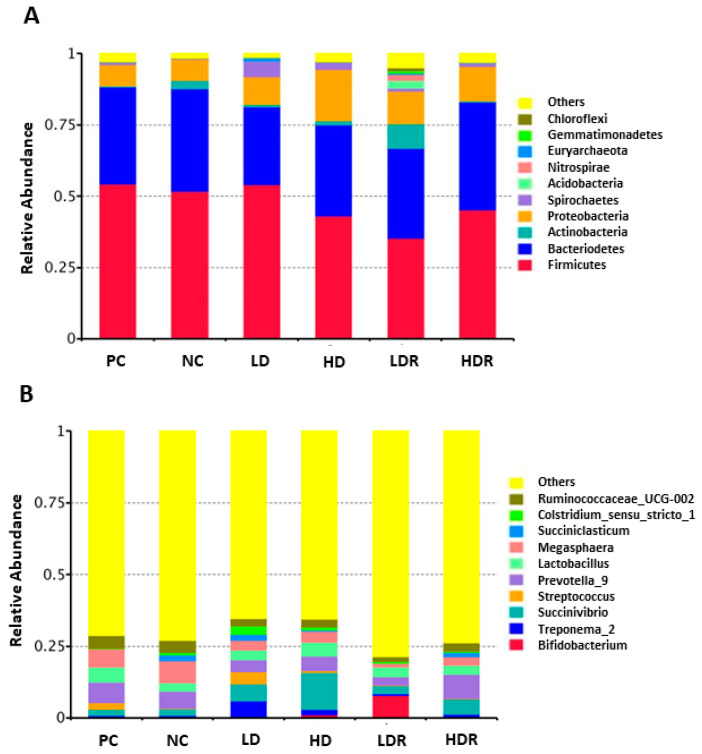
The fecal bacterial composition in pigs fed with low-protein diets supplemented with a corn-expressed phytase. (**A**) the relative abundance of bacterial community composition at phylum level, (**B**) the relative abundance of bacterial community composition at genus level. Pigs are grouped based on their dietary treatments: PC (positive control): normal protein, adequate Ca and available phosphorous (aP), no corn-expressed phytase (CEP) added; NC (negative control): low protein, adequate Ca and aP, no CEP added; LD: NC + CEP added at low dose, i.e., 2000 FTU/kg of diet; HD: NC + CEP added at high dose, i.e., 4000 FTU/kg of diet; LDR: LD with reduced calcium (Ca) and phosphorus (P); HDR: HD with reduced Ca and P. Only the top 10 phyla and genera are shown for clarity. *n* = 6 for each dietary group.

Overall, the three main phyla in all dietary groups were Firmicutes, Bacteriodetes, and Proteobacteria (Figure 4A). The most abundant bacterial community at phylum level for all six groups was Firmicutes (Figure 4A). At genus level, *Succinivibrio, Prevotella*, *Lactobacillus*, *Megasphaera,* and *Ruminococcaceae-UCG-002* were the most abundant bacteria across all diets (Figure 4B). There were differential differences among groups on the abundance of *Streptococcus, Treponema, Bifidobacterium, Clostridium*, and *Succiniclasticum* (Figure 4B).

**Figure 5:**
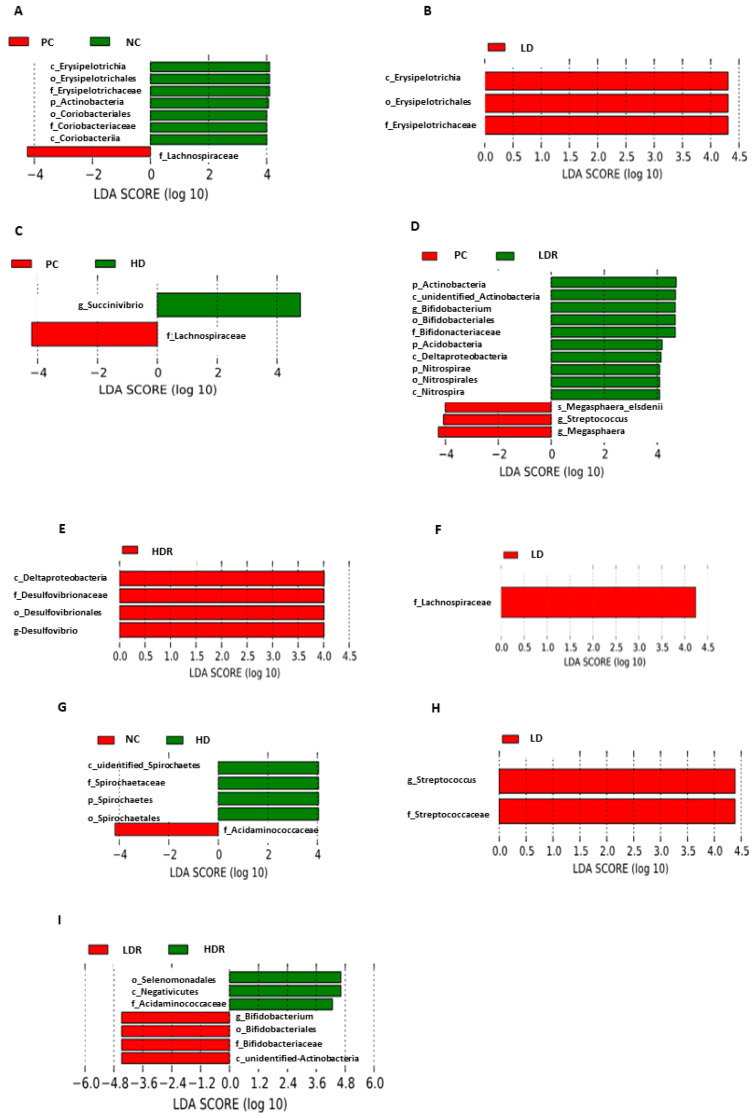
Fecal microbiota composition histograms in pigs fed with low-protein diets supplemented with a corn-expressed phytase. Histograms of linear discriminant analysis (LDA) with effect size (LEfSe) on fecal microbiota composition (**A**) PC vs. NC, (**B**) PC vs. LD, (**C**) PC vs. HD, (**D**) PC vs. LDR, (**E**) PC vs. HDR, (**F**) NC vs. LD, (**G**) NC vs. HD, (**H**) LD vs. LDR, (**I**) LDR vs. HDR. Pigs are grouped based on their dietary treatments: PC (positive control): normal protein, adequate Ca and available phosphorous (aP), no corn-expressed phytase (CEP) added; NC (negative control): low protein, adequate Ca and aP, no CEP added; LD: NC + CEP added at low dose, i.e., 2000 FTU/kg of diet; HD: NC + CEP added at high dose, i.e., 4000 FTU/kg of diet; LDR: LD with reduced calcium (Ca) and phosphorus (P); HDR: HD with reduced Ca and P. There was no significant difference on species identified for LD vs. HD and HD vs. HDR. *n* = 6 for each dietary group.

To identify the differences in gut bacterial abundances across dietary groups, LDA with LEfSe was performed (Figure 5). Pigs fed NC had a higher proportion of family Erysipelotrichaceae and family Coriobacteriaceae compared to PC that had a higher abundance of family Lachnospiraceae (LDA [log_10_] score > 2.0; Figure 5A). Pigs fed with LD had a higher abundance of family Erysipelotrichaceae compared to PC (Figure 5B). Compared to PC, the feces of HD pigs were more enriched in genus *Succinvibrio*, whereas PC pigs had a higher abundance of family Lachnospiraceae (Figure 5C). The feces of pigs fed with LDR was enriched in phylum Actinobacteria, genus *Bifidobacterium*, phylum Acidobacteria, class Deltaproteobacteria, and genus *Nitrospira,* whereas pigs fed with PC had a higher abundance of genus *Streptococcus* and genus *Megasphaera* (Figure 5D). Compared to PC, class Deltaproteobacteria and genus *Desulfovibrio* were predominant in feces of HDR pigs (Figure 5E). Compared to NC, LD had a higher abundance of family Lachnospiraceae (Figure 5F). Feces of pigs fed with HD were enriched in family Spirochaetaceae, whereas NC had a higher proportion of family Acidaminococcaceae (Figure 5G). Compared to LDR, the pigs in group LD had higher numbers of genus *Streptococcus* in their feces (Figure 5H). Pigs fed with LDR had a higher abundance of genus *Bifidobacterium* and phylum Actinobacteria compared to those fed with HDR, which had more family *Acidaminococcaceae* (Figure 5I).

## 4. Discussion

Very low-protein and P deficient diets could be potentially used to reduce the environmental concerns associated with excretion of nutrients and feed cost in swine production. However, these diets have negative influence on the growth performance and health of pigs [9,10,11,12]. The objective of the current study was to investigate whether supplementation of very low-protein, -Ca and –P diets with a CEP would improve growth, digestibility of Ca, P, and N, bone mineral density and content, serum metabolites associated with Ca and P metabolism, and fecal microbiota composition in nursery pigs. This study revealed several important findings: (1) severe reduction of dietary CP depressed the BW, ADG, ADFI, G:F and G:P; however, supplementation of these diets with CEP regardless of the doses used (i.e., 2000 and 4000 FTU/kg) with or without reduction in dietary Ca and P improved ADG and G:P ratio and tended to increase the final BW; (2) very low protein diets supplemented with CEP increased the AFD of Ca and P with or without reduction in dietary Ca and P, with a dose response of CEP on AFD of Ca and P; (3) pigs fed with very low protein diets had decreased BMD and BMC, but this decrease was completely recovered by supplementing the CEP to Ca and P-adequate diets; (4) in Ca- and P-deficient diets, supplementation of CEP at lower dose (i.e., 2000 FTU/kg of diet) did not improve the BMC, but both high (i.e., 4000 FTU/kg of diet) and low doses of added CEP completely reversed the negative effects of very low protein diets on BMD, (5) the serum concentration of osteocalcin was increased in pigs fed with very low protein diets, but supplementing these diets with CEP at 4000 FTU/kg of diet completely reversed the osteocalcin concentration in Ca- and P-deficient diet; (6) reducing the dietary CP content increased the abundance of families Erysipelotrichaceae, Acidaminococcaceae and Coriobacteriaceae, but supplementing a CEP at 2000 FTU/kg increased the family Lachnospiraceae and at 4000 FTU/kg increased the genus *Succinvibrio* in Ca and P-adequate diets. Supplementing phytase at 2000 FTU/kg in Ca and P-deficient diets increased the genus *Bifidobacterium* and phylum Actinobacteria. Overall, supplementation of very low protein diets with a CEP decreased the negative effects of these diets on average daily gain and protein efficiency ratio and increased the total tract digestibility of Ca and P regardless of the levels of Ca and P in the diet, improved bone characteristics and produced differential effects on fecal bacterial population.

The very low protein diet decreased the feed efficiency and growth performance of pigs, which was similar to previous studies [9,10,11]. The beneficial effects of microbial phytase as a feed additive on the growth performance of pigs fed with amino acids-deficient diets have been documented previously [28,29], but it is unknown whether the negative effects of low protein diets can be mitigated by using phytase produced in transgenic plants. Although both doses of CEP (i.e., 2000 and 4000 FTU/kg) showed some promising effects on certain parameters, it seems that 4000 FTU/kg CEP had added benefits. Using CEP as a supplement in very low protein diets, regardless of the doses applied, improved the ADG and G:P ratio and tended to increase the final BW; completely recovered the reduced BMD and BMC when dietary Ca and P was adequate and fully reversed the negative effects of very low protein diets on BMD when Ca and P was deficient. Compared to 2000 FTU/kg CEP, using 4000 FTU/kg CEP produced greater improvements on AFD of Ca and P, improved BMC in Ca- and P-deficient diets and completely reversed the increased serum osteocalcin concentration in Ca- and P-deficient diets when very low protein diets are fed.

For the first time, here we show that the negative outcome of protein-deficient diets on daily weight gain, final body weight and protein efficiency ratio of pigs is alleviated when these diets are supplemented with a CEP with no adverse effects of reduced dietary Ca and P. Similar positive effects for microbial phytase [20,21,22,23,24,25] and a CEP [34,35] were reported in the nursery, growing and finisher pigs fed with adequate protein but Ca and P deficient diets. The beneficial effect of CEP on the growth performance of pigs in both Ca and P adequate and deficient diets might be due to improvement in the utilization of Ca, P, and other nutrients [34,35]. Our data showed that very low protein diets supplemented with CEP at 4000 FTU/kg had a higher AFD of Ca and P. Similarly, previous studies showed that digestibility and utilization of P, Ca and CP or amino acids were increased in pigs when microbial phytase was supplemented in the diets with adequate amounts of nutrients [16,17,18]. The data from this study provide evidence that a novel CEP can be used to improve the growth performance of pigs fed with reduced CP, Ca, and P possibly through improved utilization of these nutrients.

Severe reduction of dietary CP decreased bone mass density and bone mass content. The detrimental effects of low protein diets on bone mass in humans have been reviewed previously [52]. The reduced bone mass and density in pigs fed with low protein diets might be attributed to their effects on reducing the absorption of Ca and P and secondary hyperparathyroidism [52]. This decrease in bone mass density and content was accompanied by an increased concentration of osteocalcin. Osteocalcin is a bone gamma-carboxyglutamic acid-containing protein with 49 to 50 amino acid residues produced by the osteoblast and is considered as a measure of osteoblast activity [53,54,55]. Serum osteocalcin is negatively linked with bone mineralization in pigs receiving diets with varying levels of Ca and P [56]. The high concentration of osteocalcin in pigs fed with very low protein diets may be suggestive of less available Ca and P for bone mineralization in these animals.

When very low protein diets were supplemented with a CEP, their negative effects on bone parameters were completely reversed. In parallel with these results, others reported a positive effect of CEP on bone characteristics in protein adequate, but P deficient [35] or both Ca and P deficient diets [34]. Likewise, a linear improvement in bone parameters was observed by others when microbial phytase was added to the diets of pigs with reduced Ca and P [21,24,25,57,58]. Previously, using the DXA technique, it was shown that gilts received diets with no inorganic P but supplemented with 750 FTU/kg of microbial phytase had higher BMD and BMC [23]. This positive effect on bone parameters can be due to the effect of phytase in facilitating the utilization of Ca and P, which are both required for optimal bone mineralization through building hydroxyapatite crystals [59]. In the current study, no effects of added phytase on serum Ca and P were observed; however, these diets depressed the serum concentration of osteocalcin which is suggestive of availability of Ca and P for bone mineralization [55,56,59]. Others reported a temporary drop in osteocalcin concentration in growing-finishing pigs when their diets were supplemented with a microbial phytase [60]. Our results are in line with previous reports in which no change in blood P concentration was observed when microbial phytase was used in the diet of nursery pigs [61]. However, other studies reported an increase in plasma Ca and P with supplementation of microbial phytase in diets of pigs [62,63,64].

Little is known whether the beneficial effects of CEP on growth performance are mediated by factors other than the stimulatory role of phytase in nutrients digestibility and utilization. There is some evidence on the modulatory influence of microbial phytase on intestinal microbiota in pigs [36,37], but there is a paucity of information on whether CEP can change the composition of gut organisms and hence influence the performance of pigs. This is while the importance of gut microbiota and feed efficiency in pigs has been reported [65]. The three main phyla in all dietary groups were Firmicutes, Bacteriodetes, and Proteobacteria, which is consistent with previous research where the above three populations were reported as the most dominant groups in porcine gut microbiota [66,67,68,69].

Here, for the first time, we showed that supplementing low protein diets with a CEP increased the family Lachnospiraceae and the genus *Succinvibrio* in Ca and P-adequate diets. Lachnospiraceae can produce a group of bioactive molecules that contribute to gut health [70]. Many members of this family can ferment various substrates and produce butyric acid [71]. Further, Lachnospiraceae has been reported as one of the abundant communities in pigs with the high feed conversion rate [72], and their abundance was increased in healthy pigs compared to diarrheic pigs [73]. The increased abundance of Lachnospiraceae in pigs supplemented with phytase may be suggestive of better gut health in these animals as this family may protect the pigs from pathogen infections. *Succinvibrio* are mostly localized in the colon [74] and cecum [75] in swine and have fiber-degrading potential [76]. *Succinivibrio* species have not been well studied in monogastrics, but in ruminants they are involved in hepatic gluconeogenesis via production of acetate and succinate required for propionate synthesis and improved feed efficiency [77,78]. Previous research has shown that *g. Succinivibrio* population is higher in the gut contents of pigs fed with wheat-based diet compared to those received the corn-based diet [79] and has been associated with backfat [80]. The *g. Succinivibrio* has been reported to be increased after weaning in pigs, which could be due to the availability of cereal-based diet [81]. The Lachnospiraceae family along with *Succinvibrio*, may contribute to better growth performance in pigs receiving dietary supplementation of phytase when the Ca and P level is adequate.

In the present study, supplementing a CEP in Ca and P-deficient diets increased the genus *Bifidobacterium* and phylum Actinobacteria. Bifidobacteria are saccharolytic organisms and use complex carbohydrates that escape the digestion in the proximal intestine to be able to colonize in the gastrointestinal tract [81]. The higher abundance of Bifidobacteria in the gut microbiome of pigs received supplemented CEP is suggestive of higher carbohydrate utilization capacity, which may contribute to improved performance in these pigs. The increased abundance of *Bifidobacterium* has been linked with better gut health and improved feed conversion rate in broiler chickens [82] and rats [83], which might contribute to better animal performance in Ca and P-deficient groups supplemented with CEP in the current study. Actinobacteria members are capable of secreting microbial phytase which can hydrolyze the phytic acid, release P and increase P bioavailability for the pigs [84,85]. Therefore, the increased abundance of Actinobacteria in animals fed with low protein and deficient Ca/P diets supplemented with a CEP may help the function of exogenous phytase in making the P and Ca available and improving the growth performance as well as bone parameters.

## 5. Conclusions

In summary, supplementing very low protein diets with a corn-expressed phytase decreased the negative effects of very low protein diets on average daily gain, final body weight and protein efficiency ratio, and increased the total tract digestibility of Ca and P regardless of the levels of these nutrients in the diet. Further, the negative effects of low protein diets on bone characteristics were completely recovered with supplementation of corn-expressed phytase when the dietary Ca and P levels were adequate. When dietary Ca and P were deficient, the negative effects of low protein diets on bone characteristics were improved only at higher doses of corn-expressed phytase, which was also accompanied by decreased osteocalcin concentration. Adding a corn-expresses phytase to low protein diets at a lower dose produced differential effects on the fecal bacterial population with increased family Lachnospiraceae, the genus *Bifidobacterium* and phylum Actinobacteria and at a higher dose enhanced the abundance of genus *Succinvibrio*. Thus, supplementation of very low protein diets with a corn-expressed phytase decreased the adverse effects of these diets on weight gain and protein efficiency ratio, increased the total tract digestibility of Ca and P, improved bone characteristics and produced diverse effects on fecal bacterial population. Overall, 4000 FTU/kg phytase appeared to have added benefits compared to 2000 FTU/kg phytase.

## Figures and Tables

**Table 1:** Ingredients and composition of experimental diets ^1^ (% as-fed basis)**.**

Ingredients %	Phase 2 ^2^						Phase 3 ^3^					
	PC	NC	LD	HD	LDR	HDR	PC	NC	LD	HD	LDR	HDR
Corn, yellow dent ^4^	48.49	73.77	73.71	73.67	74.23	74.2	56.8	87.1	86.95	86.89	87.57	87.52
Fish meal, menhaden ^4^	5.44	5.3	5.27	5.27	5.31	5.3	2.06	2.49	2.49	2.48	2.5	2.5
Soybean meal, 47.5% CP ^4^	35.13	3.17	3.16	3.16	3.18	3.18	38.22	4.97	4.97	4.97	5	5
Whey, dried ^4^	5.44	6.32	6.32	6.31	6.36	6.36	-	-	-	-	-	-
Corn starch ^4^	2.97	6.32	6.32	6.31	6.36	6.36	-	-	-	-	-	-
Dicalcium phosphate, 18.5% ^4^	0.7	0.95	0.95	0.95	0.13	0.13	0.98	1.18	1.18	1.17	0.38	0.38
Limestone ^4^	0.5	0.64	0.63	0.63	0.77	0.77	0.62	0.72	0.72	0.72	0.85	0.85
Vitamin premix ^5^	0.19	0.18	0.18	0.18	0.18	0.18	0.18	0.15	0.15	0.15	0.15	0.15
Trace mineral premix ^6^	0.05	0.07	0.08	0.07	0.07	0.07	0.07	0.1	0.1	0.1	0.1	0.1
Lysine, sulfate ^4^	-	1.6	1.6	1.6	1.6	1.6	-	1.58	1.57	1.57	1.57	1.57
DL-methionine ^4^	-	0.16	0.16	0.16	0.16	0.16	-	0.16	0.16	0.16	0.15	0.15
L-threonine ^4^	-	0.41	0.41	0.41	0.41	0.41	-	0.4	0.4	0.4	0.4	0.4
L-tryptophan ^4^	-	0.13	0.13	0.13	0.13	0.13	-	0.12	0.12	0.12	0.13	0.13
Salt ^4^	0.6	0.53	0.53	0.53	0.53	0.53	0.57	0.62	0.62	0.62	0.63	0.63
Chromium oxide ^4^	0.5	0.5	0.5	0.5	0.5	0.5	0.5	0.5	0.5	0.5	0.5	0.5
Grainzyme (phytase) ^4^	-	-	0.06	0.12	0.06	0.12	-	-	0.06	0.12	0.06	0.12
Calculated Chemical Composition ^7^												
Dry matter, %	90.4	90.7	90.7	90.7	90.65	90.65	89.66	89.54	89.54	89.54	89.48	89.48
Crude protein, %	24.87	13.41	13.41	13.41	13.5	13.49	24.26	12.85	12.84	12.83	12.91	12.91
Crude fat, %	3.52	3.54	3.54	3.53	3.56	3.56	3.57	3.79	3.79	3.79	3.82	3.82
Calcium, %	0.8	0.8	0.8	0.8	0.68	0.68	0.7	0.7	0.7	0.7	0.58	0.58
Phosphorus, %	0.71	0.61	0.61	0.61	0.46	0.46	0.67	0.57	0.57	0.57	0.43	0.43
aP, %	0.4	0.4	0.4	0.4	0.25	0.25	0.33	0.33	0.33	0.33	0.18	0.18
Nitrogen, %	3.98	2.15	2.15	2.15	2.16	2.16	3.88	2.05	2.05	2.05	2.06	2.06
SID Lysine	1.35	1.34	1.35	1.35	1.35	1.35	1.24	1.23	1.23	1.23	1.23	1.23
SID Methionine	0.39	0.39	0.39	0.39	0.39	0.39	0.36	0.37	0.37	0.37	0.36	0.36
SID Threonine	0.84	0.79	0.79	0.79	0.8	0.8	0.8	0.74	0.74	0.74	0.74	0.74
SID Tryptophan	0.27	0.22	0.22	0.22	0.22	0.22	0.27	0.21	0.21	0.21	0.21	0.21
Grainzyme, FTU/kg	-	-	2000	4000	2000	4000	-	-	2000	4000	2000	4000
ME, Mcal/kg	3.45	3.4	3.39	3.39	3.39	3.42	3.34	3.34	3.34	3.34	3.36	3.36
Analyzed Chemical Composition ^8^												
Dry matter, %	87.8	87.8	88.2	87.6	88.2	86.9	87.3	87	87.4	87.2	87.7	86.5
Crude protein, %	24.2	13.6	13.5	13.7	13.4	14	23.1	12	11.9	12.2	13.2	11.6
Crude fat, %	2.2	2.9	2.6	3	3	3	2.4	2.7	2.8	2.5	2.8	3.1
Chromium, %	0.21	0.23	0.22	0.2	0.27	0.26	0.23	0.2	0.2	0.2	0.24	0.21
Calcium, %	0.86	0.63	0.79	0.75	0.56	0.63	0.69	0.77	0.6	0.69	0.63	0.59
Phosphorus, %	0.74	0.6	0.61	0.58	0.42	0.43	0.63	0.58	0.52	0.58	0.46	0.43
Nitrogen, %	3.9	2.2	2.2	2.2	2.2	2.2	3.7	1.9	1.9	2	2.1	1.9

^1^ PC (positive control): normal protein, adequate Ca and available phosphorous (aP), no corn-expressed phytase (CEP) added; NC (negative control): low protein, adequate Ca and aP, no CEP added; LD: NC + CEP added at low dose, i.e., 2000 FTU/kg of diet; HD: NC + CEP added at high dose, i.e., 4000 FTU/kg of diet; LDR: LD with reduced calcium (Ca) and phosphorus (P); HDR: HD with reduced Ca and P. ^2^ Fed for two weeks of nursery phase, from day 7 to 21 (from 28 days of age and 7–11 kg body weight). ^3^ Fed for three weeks of nursery phase, from day 21 to 42 (from 42 days of age and 11–25 kg body weight). ^4^ Corn, fish meal, soybean meal, whey, corn starch, dicalcium phosphate, limestone, and salt were obtained from Nutra Blend, LLC (Neosho, MO). DL-methionine (MetAMINO^®^) and lysine, sulfate (Biolys^®^) were obtained from Evonik (Kennesaw, GA). L-threonine and L-tryptophan were obtained from Ajinomoto (Overland Park, KS). Grainzyme was obtained from Agrivida (Woburn, MA). ^5^ Vitamin premix was purchased from Ralco Animal nutrition (Marshal, MN). Each kilogram of mix contained: Vitamin A, 22,044 IU; vitamin D, 3330 IU; vitamin E, 143 IU; vitamin K, 8.83 mg; vitamin B6, 2.75 mg; vitamin B12, 18.50 mg; niacin, 99.33 mg; pantothenic acid, 90.50 mg; riboflavin 19.86 mg, Thiamine 4.41 mg; Folic Acid 2.42 mg. ^6^ Trace mineral premix was purchased from Nutra Blend, LLC (Neosho, MO). Each bag (22.68 kg) of mix contained: Iron, 7.3%; zinc, 7.3%; manganese, 2.2%; copper, 1.1%; iodine, 198 ppm; selenium, 198 ppm. ^7^ Values were calculated using National Swine Nutrition Guide (NSNG; V 2.0). ^8^ Diets chemical composition was analyzed by ServiTech (Dodge city, KS).

**Table 2:** Overall growth performance and bone minerals of pigs fed with low-protein diets supplemented with a corn-expressed phytase.

Item	Diets ^1^						SEM ^2^	*p*-Value
	PC	NC	LD	HD	LDR	HDR		
Initial BW, kg	9.85 ± 2.02	10.21 ± 1.39	10.15 ± 1.66	10.32 ± 0.99	10.10 ± 0.58	10.44 ± 2.23	0.21	0.56
Final BW, kg	28.60 ± 2.40 ^a^	14.87 ± 2.68 ^b^*^#^	18.73 ± 3.82 ^b^*	17.53 ± 1.13 ^b^	17.82 ± 2.02 ^b^	18.80 ± 3.76 ^b#^	0.73	<0.01
ADG ^3^, g/d	669 ± 81 ^a^	166 ± 80 ^c^*	306 ± 101 ^b^	257 ± 53 ^bc^	276 ± 78 ^bc^*	299 ± 80 ^b^	26	<0.01
ADFI ^3^, g/d	953 ± 64 ^a^	602 ± 174 ^b^	714 ± 20 ^b^	654 ± 141 ^b^	617 ± 118 ^b^	633 ± 48 ^b^	23	<0.01
G:F ^3^, g/g	0.70 ± 0.13 ^a^	0.28 ± 0.11 ^b^*^#$^	0.43 ± 0.14 ^b^*	0.39 ± 0.09 ^b^	0.45 ± 0.12 ^b#^	0.47 ± 0.08 ^b$^	0.02	<0.01
G:P ^3^, g/g	3.05 ± 0.42 ^ab^	2.25 ± 0.67 ^a^*	3.50 ± 1.12 ^ab^*	3.31 ± 1.09 ^ab^	3.31 ± 1.21 ^ab^	3.91 ± 0.79 ^b^	0.15	0.03
BMD ^4^, g/cm ^2^	0.64 ± 0.06 ^ac#^	0.53 ± 0.03 ^b^*	0.61 ± 0.04 ^bc^*	0.62 ± 0.04 ^cd^	0.56 ± 0.04 ^bcd#^	0.63 ± 0.09 ^cd^	0.01	0.01
BMC ^5^, g	378.92 ± 64 ^ac^	287.33 ± 36 ^b^	371.67 ± 63 ^cd^	344.14 ± 57 ^bc^	292.02 ± 19 ^b^	326.84 ± 22 ^bc^	9.21	<0.01

^1^ PC (positive control): normal protein, adequate Ca and available phosphorous (aP), no corn-expressed phytase (CEP) added; NC (negative control): low protein, adequate Ca and aP, no CEP added; LD: NC + CEP added at a low dose, i.e., 2000 FTU/kg of diet; HD: NC + CEP added at high dose, i.e., 4000 FTU/kg of diet; LDR: LD with reduced calcium (Ca) and phosphorus (P); HDR: HD with reduced Ca and P. Values are means ± standard deviations. *n* = 8 for each dietary group. ^2^ SEM: standard errors of means. ^3^ ADG: average daily gain; ADFI: average daily feed intake; G:F: gain:feed ratio; G:P: gain:protein ratio. ^4^ BMD: bone mineral density. ^5^ BMC: bone mineral content. ^a–d^ Within a row, values without a common superscript letter differ (*p* ≤ 0.05). *^#$^ Within a row, values with a common superscript symbol tend to be different (0.05 < *p* ≤ 0.1).

**Table 3:** Weekly growth performance of pigs fed with low-protein diets supplemented with a corn-expressed phytase.

Item	Diets ^1^						SEM ^2^	*p*-Value
	PC	NC	LD	HD	LDR	HDR		
BWG ^3^, g								
Wk 1	3242 ± 1515 ^a^	851 ± 511 ^b^	2157 ± 1157 ^ab^	1192 ± 872 ^b^	1475 ± 1022 ^b^	1232 ± 934 ^b^	185	<0.001
Wk 2	4994 ± 741 ^a^	1021 ± 630 ^b^	1589 ± 594 ^bc^	1702 ± 321 ^bc^	1702 ± 1131 ^bc^	2334 ± 1241 ^c^	220	<0.001
Wk 3	4994 ± 945 ^a^	1646 ± 415 ^a^	2724 ± 1372 ^b^	1816 ± 908 ^b^	1645 ± 1110 ^b^	1816 ± 1112 ^b^	224	<0.001
Wk 4	5513 ± 714 ^a^	1135 ± 1864 ^b^	2100 ± 1349 ^b^	2497 ± 1329 ^b^	2894 ± 1371 ^b^	2983 ± 1591 ^b^	278	<0.001
CFI ^3^, g								
Wk1	5714 ± 80 ^a^	3746 ± 1063^b#^	4665 ± 158 ^b#$^	3950 ± 799 ^b^	3768 ± 688 ^b$^	4190 ± 375 ^b^	135	<0.001
Wk 2	6557 ± 458 ^a^	3995 ± 1183 ^b^	4937 ± 169 ^b^	4461 ± 928 ^b^	4256 ± 785 ^b^	4404 ± 376 ^b^	161	<0.001
Wk 3	6726 ± 550 ^a^	4222 ± 1317 ^b^	4880 ± 105 ^b^	4574 ± 1014 ^b^	4381 ± 956 ^b^	4248 ± 324 ^b^	171	<0.001
Wk 4	7686 ± 897 ^a^	4903 ± 1363 ^b^	5522 ± 417 ^b^	5335 ± 1481 ^b^	4881 ± 942 ^b^	4884 ± 631 ^b^	203	<0.001
CPI ^3^, g								
Wk 1	1378 ± 18 ^a^	509 ± 144 ^b^	630 ± 21 ^b^	541 ± 109 ^b^	516 ± 94 ^b^	587 ± 52 ^b^	44	<0.001
Wk 2	1491 ± 93 ^a^	479 ± 142 ^b^	588 ± 20 ^b^	544 ± 113 ^b^	562 ± 103 ^b^	511 ± 43 ^b^	51	<0.001
Wk 3	1526 ± 113 ^a^	507 ± 158 ^b^	581 ± 12 ^b^	558 ± 123 ^b^	578 ± 126 ^b^	493 ± 37 ^b^	53	<0.001
Wk 4	1716 ± 149 ^a^	588 ± 163 ^b^	657 ± 49 ^b^	651 ± 180 ^b^	644 ± 124 ^b^	567 ± 73 ^b^	59	<0.001
G:F ^3^, g/g								
Wk 1	0.56 ± 0.26 *	0.23 ± 0.16 *	0.46 ± 0.25	0.30 ± 0.24	0.39 ± 0.23	0.29 ± 0.21	0.36	0.10
Wk 2	0.76 ± 0.15 ^a^	0.26 ± 0.15 ^b^	0.32 ± 0.12 ^b^	0.38 ± 0.14 ^b^	0.40 ± 0.23 ^b^	0.53 ± 0.25 ^ab^	0.35	<0.001
Wk 3	0.74 ± 0.14 ^a^	0.40 ± 0.09 ^b^	0.56 ± 0.27 ^ab^	0.40 ± 0.27 ^ab^	0.38 ± 0.21 ^b^	0.43 ± 0.26 ^ab^	0.36	0.02
Wk 4	0.72 ± 0.13 ^a^	0.23 ± 0.25 ^bc^	0.38 ± 0.23 ^acd^	0.47 ± 0.23 ^ac^	0.59 ± 0.35 ^ad^	0.62 ± 0.24 ^ad^	0.42	<0.001
G:P ^3^, g/g								
Wk 1	2.36 ± 1.08	1.67 ± 1.23	3.42 ± 1.90	2.20 ± 1.76	2.86 ± 1.97	2.10 ± 1.52	0.24	0.36
Wk 2	3.35 ± 0.67 ^ab^	2.13 ± 1.25 ^a^	2.70 ± 1.03 ^ab^	3.13 ± 1.19 ^ab^	3.03 ± 1.96 ^ab^	4.58 ± 2.21 ^b^	0.23	0.11
Wk 3	3.27 ± 0.64	3.25 ± 0.82	4.69 ± 0.2.27	3.25 ± 2.27	2.85 ± 1.65	3.68 ± 2.26	0.26	0.43
Wk 4	3.21 ± 0.58 ^ab^	1.93 ± 3.13 ^a^	3.20 ± 1.95 ^ab^	3.84 ± 1.89 ^ab^	4.49 ± 2.69 ^b^	5.26 ± 2.14 ^b^	0.36	0.03

^1^ PC (positive control): normal protein, adequate Ca and available phosphorous (aP), no corn-expressed phytase (CEP) added; NC (negative control): low protein, adequate Ca and aP, no CEP added; LD: NC + CEP added at low dose, i.e., 2000 FTU/kg of diet; HD: NC + CEP added at high dose, i.e., 4000 FTU/kg of diet; LDR: LD with reduced calcium (Ca) and phosphorus (P); HDR: HD with reduced Ca and P. The *p* values for the overall model effects for diet, week and diet × week for BWG were < 0.01, < 0.01 and 0.03, respectively, for CFI were < 0.01, < 0.01 and < 0.01, respectively, for CPI were < 0.01, < 0.01 and < 0.01, respectively, for G:F were < 0.01, 0.01 and 0.11, respectively, and for G:P were < 0.01, 0.05 and 0.22, respectively. Values are means ± standard deviations. *n = 8* for each dietary group. ^2^ SEM standard error of the mean ^3^ BWG: body weight gain; CFI: cumulative feed intake; CPI: cumulative protein intake; G:F: gain:feed ratio; G:P: gain: protein ratio. ^a–c^ Within a row, values without a common superscript letter differ (*p* ≤ 0.05). *^#$^ Within a row, values with a common superscript symbol tend to be different (0.05 < *p* ≤ 0.1).

## Supplementary Materials

The following are available online at https://www.mdpi.com/2076-2615/10/10/1926/s1, Figure S1: Fecal rarefaction curve analysis for pigs fed with low-protein diets supplemented with a corn-expressed phytase. Table S1: Digestibility of calcium, phosphorous and nitrogen of pigs fed with low-protein diets supplemented with a corn-expressed phytase.
