# Supplementary material for: A Novel Corn-Expressed Phytase Improves Daily Weight Gain, Protein Efficiency Ratio and Nutrients Digestibility and Alters Fecal Microbiota in Pigs Fed with Very Low Protein Diets
Source: Animals (Basel). 2020 Oct 20;10(10):1926. doi: 10.3390/ani10101926 (PMC7590218; doi:10.3390/ani10101926)
Supplement: Supplementary file 1 [file animals-10-01926-s001.pdf]

*animals*

MDPI

*Article*

# A Novel Corn-Expressed Phytase Improves Daily Weight Gain, Protein Efficiency Ratio and Nutrients Digestibility and Alters Fecal Microbiota in Pigs Fed with Very Low Protein Diets

**Cedrick N. Shili, Jonathan N. Broomhead, Shelby C. Spring and Mike B. Lanahan and Adel Pezeshki**

## Supplementary Figures

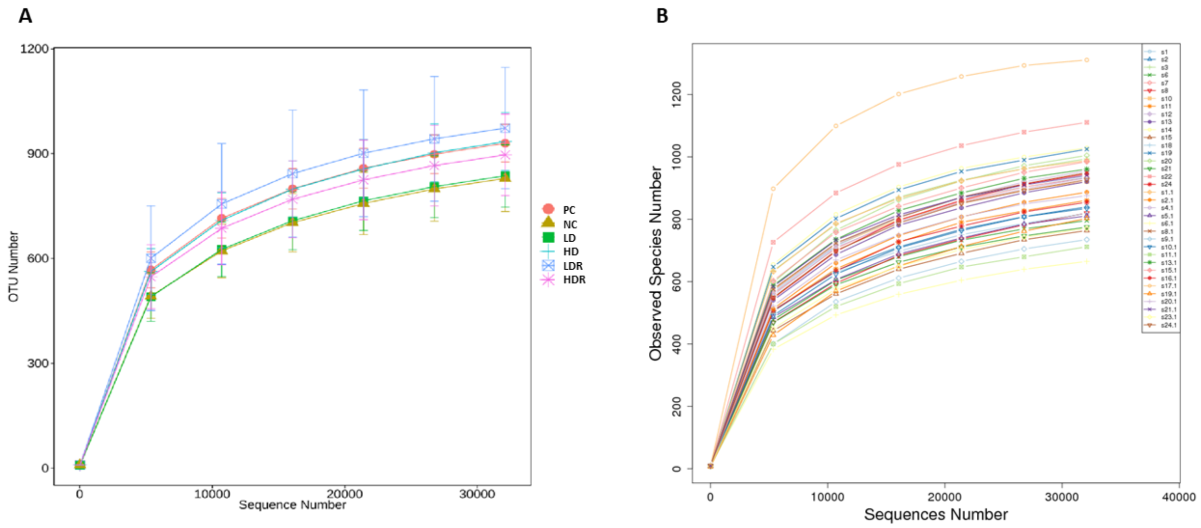


**Figure S1.** Fecal rarefaction curve analysis for pigs fed with low-protein diets supplemented with a corn-expressed phytase. The rarefaction curves from fecal samples collected show the number of operational taxonomic units (OTU) found as a function of the number of reads sampled when data were analyzed based on (**A**) dietary groups and (**B**) individual pigs. Pigs are grouped based on their dietary treatments: PC (positive control): normal protein, adequate Ca and available

animals

MDPI

phosphorous (aP), no corn-expressed phytase (CEP) added; NC (negative control): low protein, adequate Ca and aP, no CEP added; LD: NC + CEP added at low dose, i.e. 2,000 FTU/kg of diet; HD: NC + CEP added at high dose, i.e. 4,000 FTU/kg of diet; LDR: LD with reduced calcium (Ca) and phosphorus (P); HDR: HD with reduced Ca and P. Each line represents an individual pig. *n* = 6 for each dietary group.

**Table S1.** Digestibility of calcium, phosphorous and nitrogen of pigs fed with low-protein diets supplemented with a corn-expressed phytase.

| | Diets [1] | | | | | | SEM [2] | *p* - value |
|---|---|---|---|---|---|---|---|---|
| | PC | NC | LD | HD | LDR | HDR | | |
| **Calcium** | | | | | | | | |
| AFD [3], % | 65.59 ± 10.34 [a] | 67.38 ± 8.39 [ab] | 75.71 ± 7.94 [abc] | 80.82 ± 5.23 [c] | 76.03 ± 4.68 [bc] | 80.97 ± 8.53 [c] | 1.41 | < 0.01 |
| **Phosphorus** | | | | | | | | |
| AFD [3], % | 55.95 ± 9.05 [a] | 62.21 ±9.82 [ab] | 69.90 ± 9.55 [bcd] | 80.21 ± 4.44 [d] | 69.42 ± 6.63 [bd] | 75.87 ± 7.04 [cd] | 1.67 | < 0.01 |
| **Nitrogen** | | | | | | | | |
| AFD [3], % | 80.42 ± 6.32 | 74.50 ± 9.99 | 78.61 ± 4.83 | 82.23 ± 3.74 | 73.98 ± 8.53 | 77.70 ± 7.57 | 1.11 | 0.22 |

[1] PC (positive control): normal protein, adequate Ca and available phosphorous (aP), no corn-expressed phytase (CEP) added; NC (negative control): low protein, adequate Ca and aP, no CEP added; LD: NC + CEP added at a low dose, i.e. 2,000 FTU/kg of diet; HD: NC + CEP added at high dose, i.e. 4,000 FTU/kg of diet; LDR: LD with reduced calcium (Ca) and phosphorus (P); HDR: HD with reduced Ca and P. Values are means ± standard deviations. *n* = 8 for each dietary group. [2] SEM: standard errors of means; [3] AFD: apparent fecal digestibility; [a-d] Within a row, values without a common superscript letter differ ($p \leq 0.05$).
